# Internet-based cognitive behavior therapy for problem gambling in routine care: protocol for a non-randomized pilot and feasibility trial

**DOI:** 10.1186/s40814-020-00647-5

**Published:** 2020-07-21

**Authors:** Olof Molander, Philip Lindner, Jonas Ramnerö, Johan Bjureberg, Per Carlbring, Anne H. Berman

**Affiliations:** 1 Center for Psychiatry Research, Department of Clinical Neuroscience, Karolinska Institutet and Stockholm Health Care Services, Region Stockholm, Stockholm, Sweden; 2 Stockholm Centre for Dependency Disorders, Stockholm Health Care Services, Region Stockholm, Stockholm, Sweden; 3 Department of Psychology, Stockholm University, Stockholm, Sweden; 4 Department of Public Health Sciences, Stockholm University, Stockholm, Sweden; 5 Department of Psychology, Uppsala University, Uppsala, Sweden

**Keywords:** iCBT, Gambling, Problem gambling, Gambling disorder, Psychiatric comorbidity, Ordinary addiction care

## Abstract

**Background:**

Problem gambling and gambling disorder are major public health concerns worldwide, and awareness of associated negative consequences is rising. In parallel, treatment demand has increased, and Internet interventions offer a promising alternative for providing evidence-based treatment at scale to a low cost.

**Method:**

We developed a novel Internet-delivered cognitive behavioral treatment for gambling, based on qualitative interviews with treatment-seeking gamblers, behavioral research on gambling behavior, and the pathway model for problem gambling. This research protocol describes a non-randomized pilot and feasibility trial conducted in routine addiction care with adult treatment-seeking patients (max *N* = 25) with problem gambling. The primary aim is to ensure acceptability and safety, measured by satisfaction, credibility, working alliance, and possible negative effects. Secondary aims are feasibility of study procedures in terms of recruitment and measurement procedures as well as potential effectiveness measured weekly by gambling symptoms as primary outcome and gambling behavior, quality of life, symptoms of depression and anxiety, alcohol, and drug use as secondary outcomes. Potential mediators measured weekly are loss of control, verbal rules, and well-being.

**Discussion:**

This study is innovative in several respects, regarding both treatment development and implementation. The results of the study will guide a future randomized controlled trial, as well as the development of the intervention and intervention implementation within ordinary addiction care.

**Trial registration:**

Clinical trials.gov, NCT ID: NCT03946098. Registered 10 May 2019

## Background

Gambling, an activity where something of value is risked and the probability of winning or losing is less than certain [1], can lead to significant and social harm, here termed problem gambling. Problem gambling is defined as “excessive gambling behaviour that creates negative consequences for the gambler, others in his/her social network, and for the community” [2].

Problem gambling can also be expressed in terms of a clinical diagnosis. In the Diagnostic and Statistical Manual of Mental Disorders, 5th edition (DSM-5) [3], the clinical diagnostic criteria for pathological gambling were revised and labeled gambling disorder, adding gambling to addiction disorders instead of the previous diagnostic categorization as an impulse control disorder. Gambling disorder was thereby the first addictive behavior not involving a psychoactive substance to be recognized as an addiction diagnosis. Henceforth, the term problem gambling will be used here, sometimes referring to problem gamblers who also fulfil the diagnostic criteria for gambling disorder. The transferral of the gambling disorder diagnosis to addictions was associated with a political process in Sweden, whereby problem gambling received more highly profiled attention from government authorities such as the Public Health Agency and the National Board of Health and Welfare, yielding prevention research summaries and treatment recommendations presented at local and national conferences [4]. In parallel, two significant pieces of legislation were enacted in Sweden following a long period of preparation; one concerned obligatory provision of treatment on par with other addictive disorders, and the second re-regulated the gambling market, including prescription of duty of care actions on the part of the gambling industry.

Problem gambling is associated with severe negative consequences for financial and mental health, including high rates of suicidal ideation and attempts [5, 6]. The past year prevalence of problem gambling in the general population varies globally between 0.3% and 5.3% [7]. In Sweden, the estimated population prevalence of current problem gambling is 2.1% (95% confidence interval [CI] 1.8, 2.4) [8]. A meta-analysis focusing on the prevalence of problem gambling in clinical samples of treatment-seeking substance users found that approximately 23% suffered from conditions along the spectrum of problem gambling [9]. Preliminary studies have suggested that the prevalence of problem gambling among patients and clients within the Swedish primary health care system and social services is 6% and 19%, respectively [10, 11].

Meta-analyses have concluded that psychological treatments, mainly cognitive behavioral therapy (CBT), are effective for reducing gambling behavior and related problems [12, 13]. For example, Pallesen et al. concluded in one meta-analysis [13] that a range of different self-report measures had been used as outcomes in the included studies, but that the overall between-group effect size, represented as the difference between the mean score in a treatment condition and a no-treatment control condition, was 1.59 (*p* < .01) at treatment follow (averaging 17 months). Furthermore, CBT has also shown promising results in Internet-delivered formats (iCBT [14, 15];), although a recent scoping review concluded that the current literature is sparse and more research is needed [16].

Despite relatively high prevalence and associated negative consequences, problem gambling often goes untreated within routine health care. Only 10% of those afflicted seek face-to-face help for gambling problems [17]. A review of barriers for seeking help for gambling problems found that common reported reasons for not seeking treatment included a wish to handle problem by oneself, shame/embarrassment/stigma, unwillingness to admit the problem, concerns about treatment content and quality, lack of knowledge about treatment availability, or practical issues around attending treatment [18]. From a treatment-oriented perspective, the research field of problem gambling has been described as 20–30 years behind that of other substance use disorders [19, 20]. Another treatment-related challenge for problem gambling is psychiatric comorbidity. Håkansson et al. [21] found that 58% of Swedish treatment-seeking gamblers also suffered from comorbid conditions. Most common were anxiety, stress-related and somatoform disorders (29%), mood disorders (22%), and alcohol and drug use disorders (12%); this result is congruent with international research on comorbidity among treatment-seeking gamblers [22, 23]. In an effort to explain the high prevalence of comorbidity among gamblers, Blaszczynski and Nower [2] proposed a theoretical etiological pathway model with three different types of problem gamblers: behaviorally conditioned gamblers who gamble due to excitement/arousal, irrational beliefs, habituation, and chasing of losses; emotional vulnerable gamblers who gamble due to mood disturbances, life stresses, poor coping/problem-solving, and substance use; and antisocial/impulsive gamblers who gamble due to neuropsychological impulsive traits, substance use disorders, and antisocial behavior.

Although iCBT has been recommended as a suitable option, particularly for reducing barriers to accessing professional help [16], few attempts have been made to implement iCBT for problem gambling within existing addiction treatment services. We have developed a novel Internet-delivered cognitive behavioral treatment for gambling based on qualitative interviews with treatment-seeking gamblers (unpublished data), basic research on the learning and maintenance processes of gambling behavior [24], as well as the pathway model [2]. This protocol describes the first iCBT pilot and feasibility trial within a research program aiming to evaluate and implement treatment models for problem gambling within routine care.

### Objectives

The primary aim of this non-randomized pilot and feasibility trial is to ensure that a novel iCBT treatment is acceptable and safe for patients in routine care. Specifically, we aim to evaluate acceptability and safety in terms of the following:

- Treatment satisfaction
- Possible negative effects due to psychological treatment
- Treatment credibility
- Working alliance

Secondly, we aim to evaluate feasibility of the following study procedures for a future randomized controlled trial:

- Recruitment procedures and recruitment rates
- Measurement procedures within the newly developed *Stöd och Behandling* (support and treatment) (SaT) platform

Thirdly, we aim to evaluate potential effectiveness and possible mediators of treatment:

- Gambling symptoms as the primary outcome
- Gambling behavior, quality of life, symptoms of depression and anxiety, alcohol, and drug use as secondary outcomes
- Use of stimulus control strategies, loss of control in gambling situations, problematic gambling-related thinking, and well-being, all as possible mediators of treatment effects

## Methods

### Study design

The study is a non-randomized pilot and feasibility trial of iCBT with treatment-seeking participants (max *N* = 25) conducted in routine care. Participants will be assessed for diagnostic criteria of gambling disorder and psychiatric comorbidity prior to treatment and complete self-reported outcome measures pre-treatment (clinical assessment), weekly during treatment, post-treatment, and at a 3-month follow-up. Participants will be allowed 16 weeks to complete the treatment program.

The study was pre-registered at clinicaltrials.gov (NCT ID: NCT03946098). The TREND statement guidelines for non-randomized interventions [25] will be followed when reporting the trial.

### Recruitment procedure

**Fig. 1:**
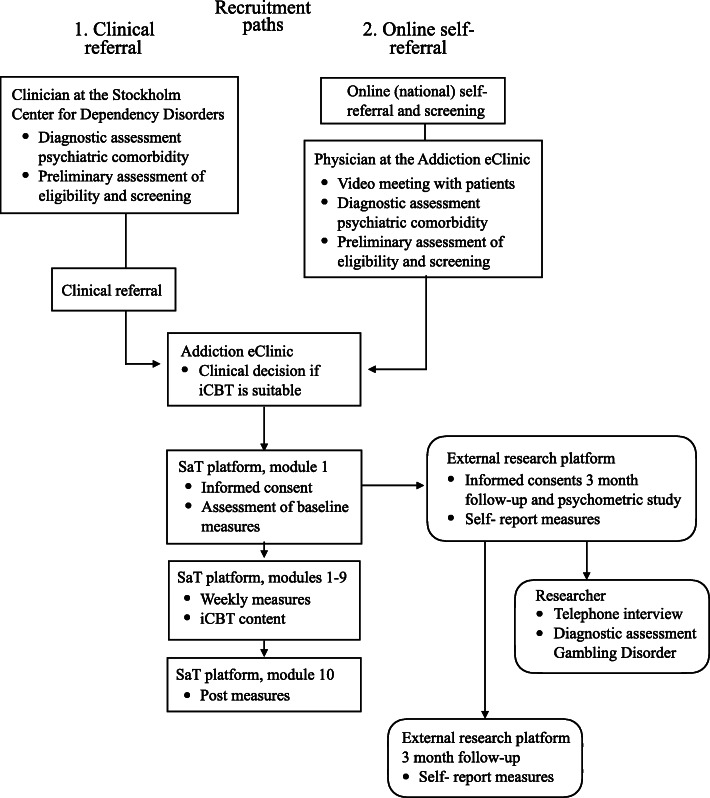
Recruitment paths and participant flow

Participants, all treatment-seeking patients, will be recruited via two paths: one indirect and the other direct. The indirect path involves recruiting patients from one of eight outpatient clinics belonging to the publicly operated Stockholm Center for Dependency Disorders. These clinics offer face-to-face treatment to patients with substance use disorders such as alcohol and drugs as well as problem gambling. Support is also offered to the patients’ concerned significant others. Patients seeking treatment for problem gambling undergo a routine assessment visit with a physician, nurse, or psychologist, on which occasion they will be informed that available treatments include face-to-face treatment or iCBT. Written information will be supplied in the form of a pamphlet, and the clinician will be able to answer any questions that arise. The recruiting clinician will make a preliminary assessment of eligibility, administrating the Problem Gambling Severity Index [26] included in the pamphlet; the purpose of this screening is to ensure that gambling symptoms have been present the last year, and these results will not be reported in the study. Patients deemed eligible and indicating interest in iCBT will be referred via formal clinical referral to the Addiction eClinic, which will make the final decision as to whether iCBT is suitable. The direct recruitment path involves recruiting participants among treatment-seeking patients accessing online self-referral to iCBT at the Addiction eClinic. A physician will screen all self-referrals and schedule suitable patients for a video conference session, for clinical assessment, after which the eClinic will make a final decision as to whether iCBT is suitable. See Fig. 1 for recruitment paths and participant flow.

### Eligibility criteria

This is a pilot and feasibility trial conducted in routine care. Although we expect that most participants will fulfill the diagnostic criteria for gambling disorder, as well as criteria for other common psychiatric comorbidities, clinical eligibility criteria will apply, where all treatment-seeking gamblers presenting problem gambling symptoms during the past year, and deemed eligible, will be offered iCBT. The inclusion criteria are (a) having a total score of > = 1 on the Problem Gambling Severity Index [26], (b) being at least 18 years old, (c) living in Sweden, (d) having the ability to work with online treatment material by themselves, and (e) being able to read and write Swedish.

Participants will be excluded from the study if they (a) fulfill criteria for ongoing manic episode or (b) are undergoing a parallel CBT treatment for problem gambling.

### Platform

Treatment will be delivered using the recently introduced and nationally available *Stöd och Behandling* (support and treatment, SaT) platform for Internet-delivered treatments within routine care, both psychiatric and somatic. Usage is not anonymous and requires login using a secure, bank-issued national e-identification solution. The same platform, with different interfaces, is used by patients and health care professionals. Regular licensed clinical psychologists at the eClinic will serve as therapists, involving monitoring, encouraging and praising compliance and progress, unlocking modules, and answering questions. This is typically done through asynchronous messages within the SaT platform, complemented as needed by telephone calls. Established clinical procedure will be followed, including telephone calls at specific timepoints in case of poor compliance (no logins or progress) or indications of suicidality.

### Measures

Self-rated measures will be collected using the SaT platform from inclusion to treatment termination and using a separate online research platform for the follow-up (due to clinical policy not permitting access to the SaT platform after concluded treatment). All self-report measures apart from initial screening will be completed by the participants online on their own. See Table 1 for full list of measures and the measurement points.

**Table 1:** Measures and measurement points

Measure	Assessment		Treatment				
	Screening	Diagnostic	Pre	Weekly	Additional	Post	Month 3
The Problem Gambling Severity Index (PGSI)	X						
The Structured Clinical Interview for Gambling Disorder (SCI-GD)		X^a^					
The Mini International Neuropsychiatric Interview version 7 (MINI-7)		X					
Consent			X^a^				
Demography			X^a^				
The Patient Health Questionnaire (PHQ-9)			X^a^			X	X
The Generalized Anxiety Disorder 7-item scale (GAD-7)			X^a^			X	X
The Alcohol Use Identification Test (AUDIT)			X^a^			X	X
The Drug Use Identification Test (DUDIT)			X^a^			X	X
The Gamblers Pathways Questionnaire (GPQ)			X^a^				
The World Health Organization Quality of Life 26 item version (WHOQOL-BREF)			X^a^			X	X
The Gambling Symptom Assessment Scale (G-SAS)			X^b^	X^b^		X	X
The Gambling TimeLine Follow Back (TLFB-G)				X^b^		X	X
Process measures^c^				X			
The Credibility/Expectancy questionnaire (CEQ)					X^d^		
The Working Alliance Inventory-Revised Short Version (WAI-SR)					X^e^		
The Client Satisfaction Questionnaire (CSQ)						X	X
The Negative Effects Questionnaire 20 items version (NEQ-20)						X	

^a^Measure is also part of a parallel psychometric study [27]

^b^Scored in reference to the week prior to seeking treatment

^c^ Process measures (possible mediators) adapted from item 15 in the Scale of Gambling Choices, item 6, 7, 8, and 13 in the Gambler’s Beliefs Questionnaire as well as well-being

^d^Administered at treatment module 2

^e^Administered at treatment module 3

#### Diagnostic assessment

Prior to treatment, participants will be screened with the Problem Gambling Severity Index (PGSI [26];) and offered to complete other measures targeting gambling behavior, as part of a separate, parallel Swedish psychometric study [27]. Criteria for Gambling Disorder and comorbidity will be assessed with clinical interviews: the Structured Clinical Interview for Gambling Disorder [28] and the Mini International Neuropsychiatric Interview, version 7 [29], respectively. Gambling type according to the pathway model [2] will be measured with the Gambling Pathways Questionnaire [30].

#### Acceptability and safety measures

Satisfaction with treatment and potential negative effects due to treatment will be measured with the Client Satisfaction Questionnaire—8-item version [31] and the Negative Effects Questionnaire—20-item version [32], respectively, both administered at post-treatment. Perceived credibility and expectancy will be measured with the Credibility/Expectancy questionnaire [33], administered at the end of the second introductory treatment module. Working alliance will be measured with the Working Alliance Inventory-Revised Short Version [34], at the end of the third treatment module.

#### Feasibility of study procedures

The recruitment rate will be defined as the number of participants recruited per month. This will serve as a basis for evaluating—and possibly adapting—the recruitment procedures described above, to ensure power in a planned future randomized controlled trial. Measurement procedures within the SaT platform will also be piloted during the trial, for example to ensure reliable measurement points and data extraction.

#### Primary outcome measure of potential effectiveness

The primary outcome, gambling symptoms, will be measured with the Gambling Symptom Assessment Scale which is a valid and reliable measure for assessing clinical change during treatment studies. The Gambling Symptom Assessment Scale have been evaluated in a population of treatment-seeking gamblers, compared to other gambling measures and clinical ratings administered weekly during a 12-week study period, showing excellent convergent validity, excellent internal consistency (α = 0.87), and test-retest reliability 0.56 [35]. In this study, the Gambling Symptom Assessment Scale will be administered at pre-treatment, weekly during treatment, post-treatment, and at the 3-month follow-up.

#### Secondary outcome measures

Secondary outcomes will encompass gambling behavior, mental health, substance use, and quality of life. Gambling behavior (gambling type, frequency, time spent, money wagered, sums won-lost) will be measured by the Gambling TimeLine Follow Back, a reliable and valid method for assessing gambling behavior among gamblers and problem gamblers [36, 37]. Mental health (depression and anxiety) will be measured by the 9-item Patient Health Questionnaire [38] and the 7-item Generalized Anxiety Disorder scale [39]. Alcohol use will be measured by the Alcohol Use Disorders Identification Test [40], and drug use will be measured by the Drug Use Disorders Identification Test [41]. Quality of life will be measured by the 26-item World Health Organization Quality of Life-BREF questionnaire [42].

#### Process measures (possible mediators)

This study will explore four possible mediators of treatment effects to be explored further in a planned future randomized controlled trial. Stimulus control strategies to limit access to gambling opportunities (for example, self-exclusion) will be measured by an item constructed for the purpose of this study. Loss of control in gambling situations will be measured by a revised version of item 15 from the Scale of Gambling Choices [43], and problematic gambling-related thinking (for example, “If I lose money gambling, I should try to win it back”) will be measured by revised versions of items 6, 7, 8, and 13 from the Gambler’s Beliefs Questionnaire [44].In order to facilitate ease of response and consistency, all items will be rated using a 0–100 visual analog scale, regardless of original response format. Participants will also rate general well-being using the same scale.

### Treatment

Treatment will consist of a 1 + 10 module iCBT program targeting problem gambling, newly developed by the authors (see TIDIER supplementary material). The pre-program module will contain an introduction to online treatment as well as collection of pre-treatment measures within the SaT platform. After that, participants will complete the 10 treatment modules at a pace of once a week, completing homework assignments facilitating behavior change during each week. The authors’ experience of iCBT delivery for addictive disorders in a clinical setting is that participants work at a slower pace than one module per week and are likely to need longer to complete the treatment. Hence, participants will be allowed 16 weeks to complete the program (with exceptions possible if deemed clinically appropriate), and measures will be collected alongside each module, which is unlocked at a maximum pace of once per week. During treatment, participants will have online contact via asynchronous secure messages with an assigned clinical psychologist at the Addiction eClinic.

**Fig. 2:**
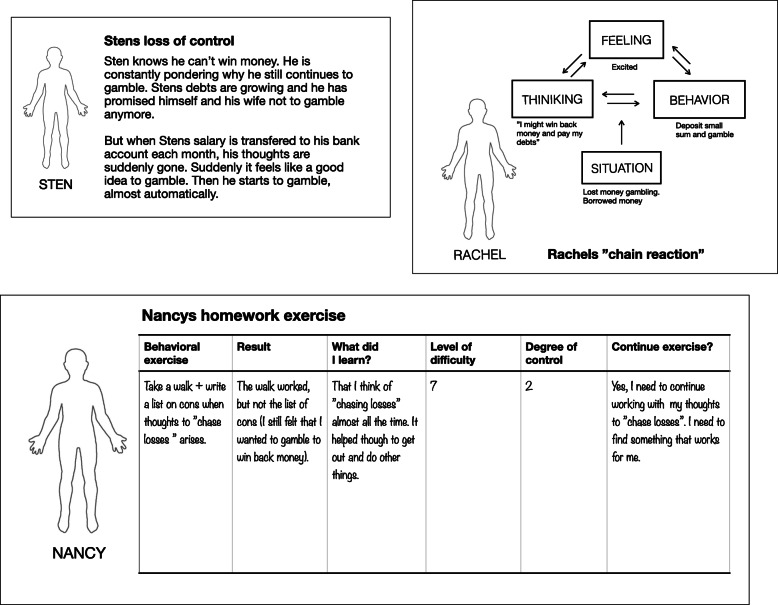
Example of treatment content. Cases illustrating treatment procedures

A bottom-up procedure was used to develop the treatment protocol, inspired by Clark’s method for developing novel CBT treatments [45]. A comprehensive description of the development process is outside the scope of this article and will be described elsewhere. Briefly, we developed a clinical model delineating factors that contribute to the persistence of problem gambling behavior. We then aligned these with treatment interventions targeting each specific factor, based on qualitative interviews with treatment-seeking gamblers with or without psychiatric comorbidity (unpublished data), basic experimental research on the learning, and maintenance processes of gambling behavior [24], as well as the pathway model [2]. See Table 2 for a description of treatment content per module and corresponding exercises, and Fig. 2 for examples of iCBT treatment content.

**Table 2:** Overview of treatment components

Module	Brief description	Content and exercises
0	Introduction to online treatment and collection of pre-measures	
1	Why problem gambling persists Presentation of clinical model	Discrimination training^a^ A first step towards behavior change
2	Loss of control in gambling situations Strategies and loss of control	Identify strategies Discrimination training^a^ A first step towards behavior change
3	Behavioral exercises	Difficulty rating of gambling situations Discrimination training^a^ Behavioral exercises targeting loss of control
4	How thoughts are affected by gambling: ‘Chasing’ and ‘autopilot’ gambling	Discrimination training^a^ Behavioral exercises targeting loss of control
5	Why gambling situations continue to be challenging: expectancy before gambling	Discrimination training^a^ Behavioral exercises targeting loss of control
6	What happens while gambling: common reactions, “the zone”	Discrimination training^a^ Behavioral exercises targeting loss of control
7	What happens while gambling: other reactions facilitating continuous gambling behavior	Discrimination training^a^ Behavioral exercises targeting loss of control
8	Further behavioral exercises	Discrimination training^a^ Behavioral exercises targeting loss of control
9	Further behavioral exercises	Discrimination training^a^ Behavioral exercises targeting loss of control
10	Treatment summary Maintenance plan Collection of post-measures	Individual evaluation and treatment summary Continuous behavioral exercises

^a^Discrimination training refers in this context to procedures aimed at present moment discriminating of antecedents and consequences of gambling related behavior

### Participant safety

This study will be conducted within routine care, meaning that all patient safety procedures apply. Before the study, all patients and potential participants are assessed for suitability for online treatment (including assessment of suicide risk), patient activity (including measures) is monitored weekly, and there is a procedure for handling absence of activity, including secure messages, telephone calls, and/or letters, at different timepoints. Patients will have the option of continuing contact with their recruiting clinic for any additional treatment needs.

### Ethical considerations

All participants will provide digital informed consent in the first pre-program module of the online program. Since the study is carried out within a clinical context with treatment-seeking patients, those patients who do not wish to provide consent for research participation will nonetheless receive exactly the same treatment, but not included in study-related data collection and analysis. This study is approved by the Regional Ethics Board of Stockholm, Sweden (ref. no. 2017/1479-31), pending a minor amendment.

## Planned statistical analyses

### Acceptability measures

Descriptive statistics on treatment credibility, treatment satisfaction, and working alliance will be presented in means and standard deviations. Occurrence of possible negative effects due to psychological treatment will be presented descriptively and in frequencies, means, and standard deviations.

### Potential effectiveness

**Fig. 3:**
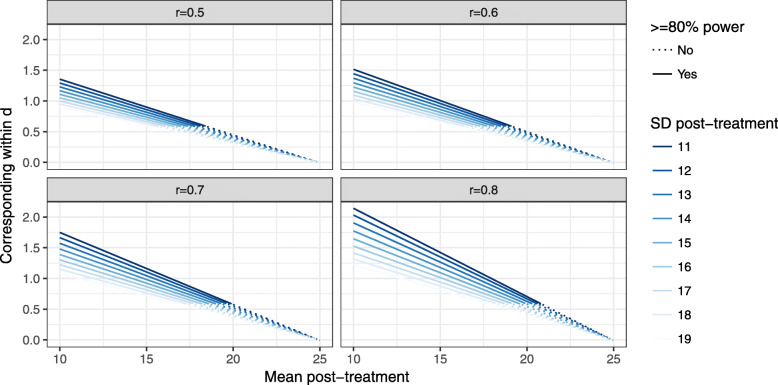
Power curves as a function of post-treatment means, SDs, and within-r, with line weights according to resulting effect size

Scores from the Gambling Symptom Assessment Scale and TLFB-derived measures, collected weekly, will be modeled using appropriate mixed effects models [46] depending on distribution (e.g., Gaussian, lognormal, Poisson, zero-inflated versions), with maximum likelihood estimation of missing data and appropriate numeric time-variable(s) [47]. Outcome measures collected less frequently will be modeled using Generalized Estimating Equations. The results will be used to calculate power and sample size in a planned future randomized controlled trial. In the current study, we aim to recruit *n* = 25 participants to assess feasibility; assuming that sample baseline means and standard deviations on the Gambling Symptom Assessment Scale are similar to an ongoing treatment study at the same treatment center (*M* = 24.9, SD = 11), the pilot study should be able to detect a within-group effect size of at least *d* > 0.6 under realistic circumstances. See Fig. 3 for a power spectrum plot, showing what corresponding effect size (as a function of a range of post-treatment means and standard deviations) can be detected with 80% power, with different assumed within-subject correlations.

We will explore possible mediators of treatment effects by examining correlations between change in symptoms and change in the process measures, and we will also explore time-lagged effects. Descriptive statistics on acceptability measures and platform activity (completed modules, number of logins, etc.) will also be presented.

## Discussion

This non-randomized trial aims to evaluate the acceptability, feasibility, potential effectiveness, and possible negative effects of a novel iCBT protocol in a sample of treatment-seeking participants with problem gambling within routine care. The study is innovative in several respects.

Firstly, the study will be conducted in routine care for problem gambling with a sample of treatment-seeking patients, most of whom are expected to meet criteria for gambling disorder. The external validity of the study will thus be very high. All treatment-seeking patients, including those with comorbidities, will be offered iCBT. This is uncommon, as patients with comorbidities are often excluded from treatment studies as well as from gambling treatment within routine care. A further advantage with iCBT is that it can reduce the waiting time to initiate treatment. The waiting time for group treatment for problem gambling at the Stockholm Center for Dependency Disorders can vary greatly between clinics and seasons: patients may have to wait between 1 and 12 months, depending on demand; such a long wait is often too long when considering treatment-relevant factors such as motivational windows that often co-occur with treatment-seeking [48].

Second, we have used a bottom-up approach in the treatment development process. This includes deriving the treatment content from in-depth clinical interviews with treatment-seeking gamblers, research on the learning, and maintenance processes of gambling behavior, as well as the pathway model for PG [2]. Also, we designed the treatment protocol to build on a simple, delimited set of interventions of presumed greatest importance. This is contrary to typical addiction treatments that offer a smorgasbord of exercises and treatment rationales in an attempt to capture all relevant aspects that may apply differently to different patients. As a research field, the study of treatment for problem gambling is still in its infancy. Current CBT protocols for problem gambling are seldom based on a functional analysis on why problem gambling behavior persists over time despite negative consequences. This is so, despite the fact that problem gambling is a phenomenon that has generated basic research on the learning processes involved. In general, a broad mixture of general CBT components, which have been found effective for other conditions such as depression, anxiety, or alcohol problems, have often been arbitrarily combined into treatment protocols [49], while interventions targeting key gambling processes such as “chasing losses,” or “loss of control” [2, 50] have been lacking. In contrast, utilizing a few, carefully selected treatment components, will hopefully enable a more clear definition of what to prioritize in treatment, opportunities for continuous applied behavior change, as well as better controlled studies in terms of mediating and moderating factors.

Third, this study aims to evaluate novel treatment content in an iCBT format without prior face-to-face evaluation. This is uncommon, but in view of the large body of evidence indicating that iCBT yields outcomes comparable to face-to-face treatment [51], the increasing acceptance of iCBT as a treatment form, and the availability of an eClinic within the Stockholm Center for Dependency Disorders as the trial setting, we have opted to eliminate the step of face-to-face evaluation, instead using telephone and video meetings, prior to the iCBT launch in this pilot and feasibility trial. Additionally, we see the use of the nationally available SaT digital platform, as enabling rapid development, deployment and dissemination, evaluation, and optimization of novel treatment interventions into routine care.

The study will also be subject to some limitations. A non-randomized pilot and feasibility trial will not yield knowledge on whether observed effects are causal, nor enable evaluation of effects for specific subgroups of gamblers through randomization to typical or subgroup-tailored treatment. By design, this single-arm study will only provide an uncontrolled estimate of the treatment effect and not of the spontaneous remission or treatment-as-usual, to which the treatment effect will be compared in future randomized trials. However, non-randomized pilot trial designs are common as a first step in the development and evaluation of novel interventions. Apart from evaluating acceptability, feasibility, possible effectiveness, and potential negative effects, the pilot outcomes of this study will hopefully provide some clues as to whether further treatment adaptations for problem gambling and comorbidity specifically, might be necessary, a research question which can then be explored in a future randomized controlled trial.

## Supplementary information

**Additional file 1.** TIDIER.

## Data Availability

No data is available.

## Abbreviations

CBT: Cognitive behavioral therapy
DSM-5: Diagnostic and Statistical Manual of Mental Disorders, 5th edition
iCBT: Internet-delivered cognitive behavioral therapy
ISRII: International Society for Research on Internet Interventions
REGAPS: Responding to and Reducing Gambling Problems–Studies in Help-seeking, Measurement, Comorbidity and Policy Impacts
SaT: The *Stöd och Behandling* (support and treatment) platform
