# Supplementary material for: Internet-based cognitive behavior therapy for problem gambling in routine care: protocol for a non-randomized pilot and feasibility trial
Source: Pilot Feasibility Stud. 2020 Jul 21;6:106. doi: 10.1186/s40814-020-00647-5 (PMC7372781; doi:10.1186/s40814-020-00647-5)
Supplement: Supplementary file 1 — Additional file 1. TIDIER. [file 40814_2020_647_MOESM1_ESM.pdf]

TIDieR

Internet-based Cognitive Behavior Therapy for Problem Gambling

Why:

We have used a bottom-up approach in the treatment development process. This includes deriving the treatment content from in-depth clinical interviews with treatment- seeking gamblers, research on the learning and maintenance processes of gambling behavior, as well as the Pathway model for problem gambling. Also, we designed the treatment protocol to build on a simple, delimited set of interventions of presumed greatest importance. This is contrary to typical addiction treatments that offer a smorgasbord of exercises and treatment rationales in an attempt to capture all relevant aspects that may apply differently to different patients. As a research field, the study of treatment for problem gambling is still in its infancy. Current cognitive behavior therapy (CBT) protocols for problem gambling are seldom based on a functional analysis on why problem gambling behavior persists over time despite negative consequences. This is so, despite the fact that problem gambling is a phenomenon that has generated basic research on the learning processes involved. In general, a broad mixture of general CBT components, which have been found effective for other conditions such as depression, anxiety or alcohol problems, have often been arbitrarily combined into treatment protocols, while interventions targeting key gambling processes such as “chasing losses”, or “loss of control” have been lacking. In contrast, utilizing a few, carefully selected treatment components, will hopefully enable a more clear definition of what to prioritize in treatment, opportunities for continuous applied behavior change, as well as better controlled studies in terms of mediating and moderating factors.

What (material):

Treatment will be delivered using the recently introduced and nationally available Swedish *Stöd och Behandling* (Support and Treatment, SaT) platform for internet-delivered treatments within routine care, both psychiatric and somatic. Usage is not anonymous and requires login using a secure, bank-issued national e-identification solution. The same platform, with different interfaces, is used by patients and health care professionals. This is typically done through asynchronous messages within the SaT platform, complemented as needed by telephone calls. Established clinical procedure will be followed, including telephone calls at specific timepoints in case of poor compliance (no logins or progress) or indications of suicidality.

What (procedures):

The treatment consist of CBT based content in the form of text and images (se below for treatment components). The full treatment protocol is in Swedish and is not yet published.

Internet based CBT for problem gambling, overview of treatment protocol

Module	Brief description	Content and exercises
0	Introduction to online treatment and collection of pre-measures	
1	Why problem gambling persists<br>Presentation of clinical model	Discrimination training[a]<br>A first step towards behavior change
2	Loss of control in gambling situations<br>Strategies and loss of control	Identify strategies<br>Discrimination training[a]<br>A first step towards behavior change
3	Behavioral exercises	Difficulty rating of gambling situations<br>Discrimination training[a]<br>Behavioral exercises targeting loss of control
4	How thoughts are affected by gambling: 'Chasing' and 'autopilot' gambling	Discrimination training[a]<br>Behavioral exercises targeting loss of control
5	Why gambling situations continue to be challenging: Expectancy before gambling	Discrimination training[a]<br>Behavioral exercises targeting loss of control
6	What happens while gambling: Common reactions, 'the zone'	Discrimination training[a]<br>Behavioral exercises targeting loss of control
7	What happens while gambling: Other reactions facilitating continuous gambling behavior	Discrimination training[a]<br>Behavioral exercises targeting loss of control
8	Further behavioral exercises	Discrimination training[a]<br>Behavioral exercises targeting loss of control
9	Further behavioral exercises	Discrimination training[a]<br>Behavioral exercises targeting loss of control
10	Treatment summary<br>Maintenance plan<br>Collection of post-measures	Individual evaluation and treatment summary<br>Continuous behavioral exercises

[a] Discrimination training refers in this context to procedures aimed at present moment discriminating of antecedents and consequences of gambling related behavior

How (mode of delivery; individual or group): The internet based CBT will be delivered individually. Regular licenced clinical psychologists at the eClinic will serve as therapists, involving monitoring, encouraging and praising compliance and progress, unlocking modules, and answering questions. During treatment, participants will have online contact via asynchronous secure messages with an assigned clinical psychologist at the Addiction eClinic.

Where: Participants will be recruited among patients at one of eight outpatient clinics belonging to the publicly operated Stockholm Center for Dependency Disorders. Patients will be informed during a ordinary visit with a physician, nurse or psychologist that available treatments include iCBT. Written information will be supplied in the form of a pamphlet, and the clinician will be able to answer any question. The recruiting clinician will make a preliminary assessment of eligibility, i.e. PGSI score, and make a formal clinical referral to the Addiction eClinic, a specialized clinic that is part of the Stockholm Center for Dependency Disorders, which will make the final decision as to whether iCBT is suitable. Since this trial is conducted within ordinary care, all patients referred to the eClinic will be offered the treatment regardless of study participation. Informed consent will be collected digitally in the first, introductory module of the online program. Only patients who consent will be included in this study; those who decline will nonetheless be offered the exact same treatment, but excluded from study-related data collection and analysis.

When and how much: Treatment will consist of a 1+10 module iCBT program targeting problem gambling. The pre-program module will contain an introduction to online treatment as well as collection of pre-treatment measures within the SaT platform. After that, participants will complete the 10 treatment modules at a pace of once a week, completing homework assignments facilitating behavior change during each week. The authors' experience of iCBT delivery for addictive disorders in a clinical setting is that participants work at a slower pace than one module per week and are likely to need longer to complete the treatment. Hence, participants will be

allowed 16 weeks to complete the program (with exceptions possible if deemed clinically appropriate), and measures will be collected alongside each module, which is unlocked at a maximum pace of once per week.

Tailoring: No tailored interventions are planned.

How well (planned): The internet delivered CBT will consist of content (words and images) in 10 modules, within the SAT platform. As such, all participants will access the same treatment content. Therapist will provide support via asynchronous secure messages, and no evaluation of therapist treatment fidelity is planned. Descriptive statistics on acceptability measures and participant platform activity (completed modules, number of logins et cetera) will be presented.
